# Supplementary material for: Memory and decision making interact to shape the value of unchosen options
Source: Nat Commun. 2021 Jul 30;12:4648. doi: 10.1038/s41467-021-24907-x (PMC8324852; doi:10.1038/s41467-021-24907-x)
Supplement: Supplementary file 1 — Supplementary Info [file 41467_2021_24907_MOESM1_ESM.pdf]

Supplementary Material

Memory and Decision Making Interact to Shape the Value of Unchosen Options

Natalie Biderman[1*] and Daphna Shohamy[1,2*]

[1]Department of Psychology and Mortimer B. Zuckerman Mind, Brain, Behavior Institute
[2]The Kavli Institute for Brain Science
Columbia University, New York, NY

Supplementary Figures
Supplementary Tables
Supplementary Text

Supplementary Figures

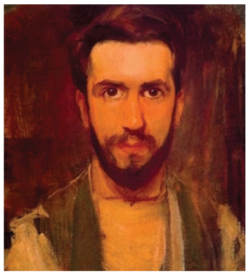

Piet Mondrian
Self Portrait
1900

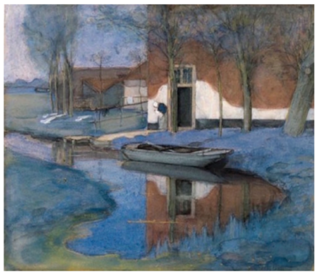

Piet Mondrian
A Farmbuilding
1900–1901

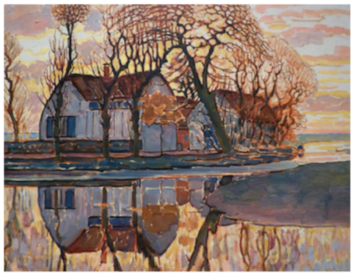

Piet Mondrian
Farm Near Duivendrecht
1916

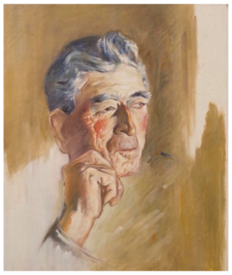

Clyfford Still
PH-269
1941

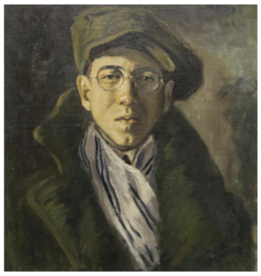

Clyfford Still
PH-672
1923

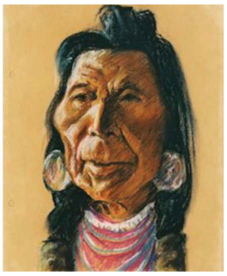

Clyfford Still
PP-241
1936

Supplementary Figure 1. Art images depicted in Figure 1. In the study, all images were converted to 300x300 pixels. Courtesy of Clyfford Still Museum, Denver, CO © 2021 City and County of Denver / ARS, NY, and the Mondrian/Holtzman Trust ©2021.

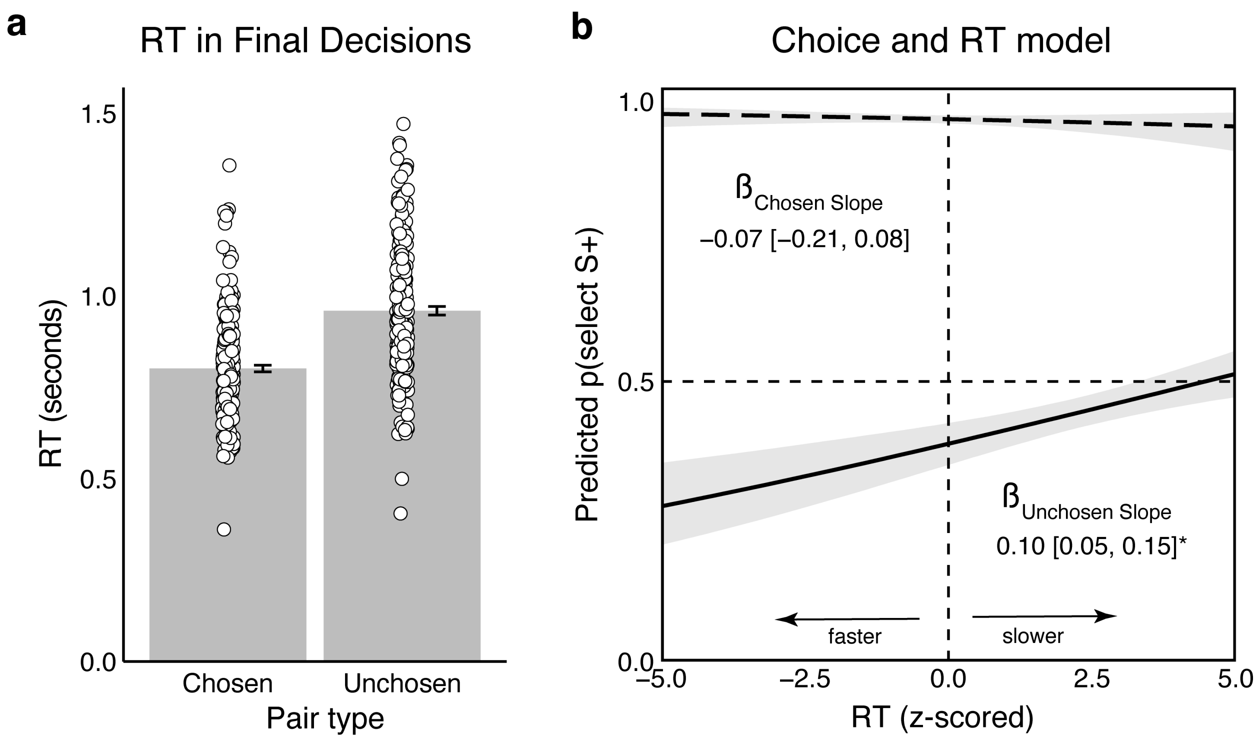

Supplementary Figure 2. Reaction times (RT) in the Final Decisions phase in Experiment 1 (n = 235). **(a)** Participants were faster to decide among chosen pairs compared to unchosen pairs. **(b)** Choices in the Final Decisions phase were modulated by reaction times in unchosen pairs, but not in chosen pairs. In unchosen pairs, participants were faster when selecting items previously paired with unrewarded items, as indicated by the substantial positive slope in a multilevel Bayesian logistic regression predicting the probability to select rewarded items as a function of pair type and normalized reaction times (using z-score). In panel **a**, error bars denote standard error of the mean and points denote trial-averaged data of individual participants. In panel **b**, the ß coefficients and model fits denote median estimation and 95% highest density interval (HDI) of the posterior distribution. Coefficients with 95% HDI that exclude zero are marked with an asterisk. S+ denotes a rewarded stimulus (for unchosen stimuli, this is the outcome of their chosen counterpart). Source data are provided as a Source Data file.

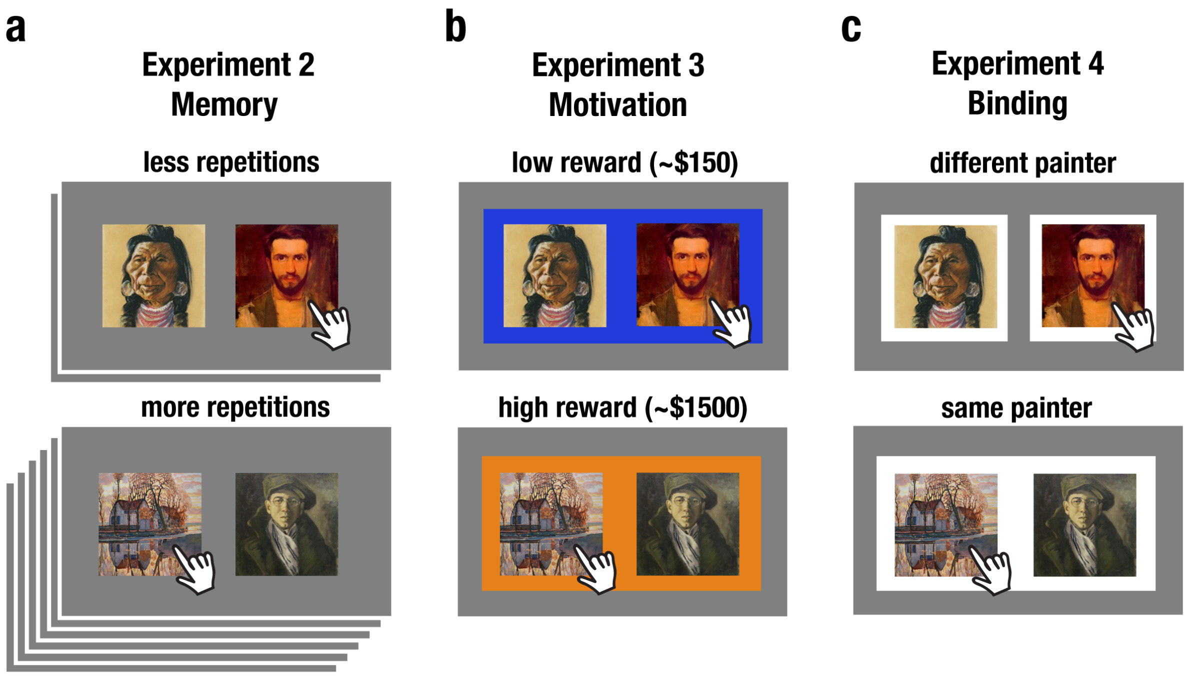

Supplementary Figure 3. Experiments 2, 3 and 4 introduced manipulations to the deliberation phase. In all experiments, participants had to deliberate and make decisions about 16 pairs of paintings. **a** Experiment 2 manipulated the number of repetitions of the pairs. Half of the pairs repeated two times and the other half repeated six times. **b** Experiment 3 manipulated the magnitude of the reward. Half of the pairs were marked as coming from a low-stakes gallery (earnings were centered around $150 and standard deviation of $10, marked with a blue frame) and the other half from a high-stakes gallery (earnings centered around $1500 and standard deviation of $100, marked with an orange frame). Participants were told that they would receive 0.2% of the earnings of one of their chosen paintings. **c** Experiment 4 manipulated the possible grouping of deliberation pairs. Half of the pairs were marked as painted by a different painter (two separate white frames) and half by the same painter (a single white frame). The art images in the figure include detail images of the artworks PH-672 (1923), and PP-241 (1936) by Clyfford Still, courtesy the Clyfford Still Museum, Denver, CO © 2021 City and County of Denver / ARS, NY, and the artworks Self Portrait (1900), and Farm Near Duivendrecht (1916) by Piet Mondrian, courtesy of Mondrian/Holtzman Trust © 2021. See Supplementary Figure 1 for the full images.

Supplementary Tables

Supplementary Table 1. Behavioral performance in all experiments.

**Exp.**	**Condition**	**S+ Selection (Chosen pairs)**	**S+ Selection (Unchosen pairs)**	**Pairs Memory**	**Choice Memory**
Pilot	-	0.92 ± 0.01	0.42 ± 0.02	0.61 ± 0.02	0.84 ± 0.02
Exp. 1	-	0.92 ± 0.01	0.43 ± 0.01	0.65 ± 0.01	0.84 ± 0.01
Exp. 2	More repetitions	0.90 ± 0.01	0.44 ± 0.02	0.66 ± 0.02	0.89 ± 0.02
	Less repetitions	0.93 ± 0.01	0.46 ± 0.02	0.60 ± 0.01	0.90 ± 0.02
Exp. 3	High reward	0.93 ± 0.01	0.44 ± 0.02	0.63 ± 0.02	0.83 ± 0.02
	Low reward	0.89 ± 0.02	0.47 ± 0.03	0.64 ± 0.01	0.84 ± 0.01
Exp. 4	Same painter	0.89 ± 0.02	0.42 ± 0.03	0.66 ± 0.02	0.81 ± 0.02
	Different painter	0.89 ± 0.02	0.48 ± 0.02	0.58 ± 0.02	0.80 ± 0.02

Note: "S+ selection" refers to the probability of selecting a rewarded painting in the Final Decisions phase, separated for chosen and unchosen pairs (for unchosen items, outcome assignment was according to their associated chosen items). "Pairs memory" refers to the accuracy measure in the memory test for the deliberation pairs in the Surprise Memory phase. "Choice memory" is the accuracy in the memory test for the chosen item in each pair in the Surprise Memory test. Table cells depict mean ± standard error of the mean. Exp. = Experiment.

Supplementary Table 2. Regression coefficients in the Final Decision phase.

**Exp.**	**Intercept**	**Choice**	**Ratings**	**Choice x Ratings**
Pilot	1.35 [1.17 1.54]*	1.80 [1.59 2.01]*	0.12 [-0.10 0.35]	-0.13 [-0.37 0.11]
Exp. 1	1.49 [1.37 1.62]*	1.91 [1.77 2.05]*	0.32 [0.19 0.45]*	-0.03 [-0.16 0.10]
Exp. 2	1.69 [1.49 1.91]*	1.95 [1.70 2.20]*	0.36 [0.21 0.51]*	-0.10 [-0.24 0.04]
Exp. 3	1.66 [1.46 1.88]*	1.92 [1.66 2.17]*	0.30 [0.13 0.46]*	-0.06 [-0.22 0.10]
Exp. 4	1.55 [1.34 1.77]*	1.84 [1.58 2.12]*	0.27 [0.17 0.36]*	-0.03 [-0.11 0.06]
**Exp.**	**Condition**	**Choice x Condition**	**Ratings x Condition**	**Choice x Ratings x Condition**
Exp. 2	-0.14 [-0.27 -0.02]*	-0.11 [-0.23 0.01]	0.03 [-0.11 0.16]	0.02 [-0.12 0.17]
Exp. 3	0.11 [-0.01 0.23]	0.21 [0.07 0.36]*	0.02 [-0.13 0.17]	0.01 [-0.15 0.17]
Exp. 4	-0.07 [-0.23 0.09]	0.05 [-0.08 0.18]	0.03 [-0.04 0.11]	0.02 [-0.05 0.10]

Note: In all experiments we ran the following Bayesian multilevel logistic regression: p(select S+) = $logit^{-1}(ß_0 + ß_1*choice + ß_2*\Delta ratings + ß_3*choice*\Delta ratings)$. For Experiment 2-4, we also included a predictor for condition and its interactions with the other predictors (Experiment 2: more vs. less repetitions, Experiment 3: high vs. low reward, Experiment 4: same vs. different painter). Columns represent the different coefficients and cells depict median and 95% highest density intervals (HDIs) of the posterior distribution of each coefficient. HDIs that exclude zero are marked with an asterisk. Exp. = Experiment.

Supplementary Table 3. Coefficients of interest for chosen and unchosen pairs separately.

**Exp.**	**Condition**	**Intercept (Chosen pairs)**	**Slope (Chosen pairs)**	**Intercept (Unchosen pairs)**	**Slope (Unchosen pairs)**
Pilot		3.14 [2.85 3.46]*	-0.01 [-0.40 0.38]	-0.45 [-0.70 -0.20]*	0.25 [-0.00 0.52]
Exp. 1		3.40 [3.18 3.63]*	0.29 [0.08 0.50]*	-0.42 [-0.56 -0.27]*	0.35 [0.19 0.51]*
Exp. 2	More repetitions	3.39 [2.99 3.82]*	0.31 [0.01 0.61]*	-0.29 [-0.58 -0.02]*	0.46 [0.23 0.70]*
	Less repetitions	3.89 [3.45 4.39]*	0.21 [-0.15 0.55]	-0.22 [-0.47 0.03]	0.45 [0.24 0.67]*
Exp. 3	High reward	3.90 [3.44 4.42]*	0.27 [-0.13 0.67]	-0.35 [-0.64 -0.06]*	0.37 [0.11 0.63]*
	Low reward	3.25 [2.86 3.69]*	0.20 [-0.11 0.53]	-0.14 [-0.45 0.16]	0.34 [0.07 0.61]*
Exp. 4	Same painter	3.36 [2.93 3.86]*	0.29 [0.10 0.50]*	-0.40 [-0.73 -0.09]*	0.30 [0.16 0.45]*
	Different painter	3.40 [2.94 3.93]*	0.18 [0.00 0.37]*	-0.17 [-0.43 0.09]	0.29 [0.15 0.43]*

Note: By design, chosen and unchosen pairs included different value signals (outcomes were explicit for chosen pairs but could only be inferred for unchosen pairs), so we rearranged the model coefficients (see Supplementary Table 2) to generate two measures of interest for chosen and unchosen pairs: (1) slope: the influence of subjective ratings on choice (e.g., for Experiment 1, $ß = ß_2 + ß_3$*choice), and (2) intercept: the tendency to choose gain paintings when the two options do not differ in their subjective ratings (e.g., for Experiment 1, $ß = ß_0 + ß_1$*choice). Columns represent the different coefficients and cells depict median and 95% highest density intervals (HDIs) of the posterior distribution of each coefficient. HDIs that exclude zero are marked with an asterisk. Exp. = Experiment.

Supplementary Table 4. Coefficients of interest for chosen and unchosen pairs across conditions.

Exp.	Intercept (Chosen pairs)	Slope (Chosen pairs)	Intercept (Unchosen pairs)	Slope (Unchosen pairs)
Exp. 2	3.64 [3.25 4.06]*	0.26 [0.03 0.49]*	-0.25 [-0.46 -0.05]*	0.46 [0.29 0.62]*
Exp. 3	3.58 [3.19 3.99]*	0.24 [-0.02 0.50]	-0.25 [-0.48 -0.01]*	0.36 [0.16 0.55]*
Exp. 4	3.39 [2.97 3.84]*	0.24 [0.10 0.38]*	-0.28 [-0.50 -0.07]*	0.30 [0.18 0.42]*

Note: The coefficients ignore variability caused by condition type (condition is set to 0 in the model). Columns represent the different coefficients and cells depict median and 95% highest density intervals (HDIs) of the posterior distribution of each coefficient. HDIs that exclude zero are marked with an asterisk. Exp. = Experiment.

Supplementary Table 5. Pairs memory and decision bias model regression coefficients.

Exp	Intercept	Memory	Condition	Memory x Condition
Pilot	0.14 [-0.06, 0.35]	0.59 [0.27, 0.91]*	-	-
Exp. 1	0.06 [-0.08, 0.20]	0.67 [0.47, 0.88]*	-	-
Exp. 2	0.04 [-0.14, 0.21]	0.68 [0.40, 0.96]*	-0.09 [-0.26, 0.08]	0.11 [-0.16, 0.37]
Exp. 3	-0.04 [-0.23, 0.14]	0.78 [0.49, 1.07]*	0.01 [-0.17, 0.19]	0.04 [-0.24, 0.31]
Exp. 4	-0.05 [-0.18, 0.09]	0.76 [0.55, 0.98]*	-0.19 [-0.32, -0.05]*	0.30 [0.10, 0.51]*
**Exp.**	**Memory (Condition1)**	**Memory (Condition2)**		
Exp. 2	0.79 [0.45, 1.13]*	0.57 [0.14, 1.00]*		
Exp. 3	0.82 [0.45, 1.18]*	0.75 [0.33, 1.18]*		
Exp. 4	1.07 [0.78, 1.35]*	0.46 [0.15, 0.77]*		

Note: In all experiments we ran a Bayesian linear regression predicting inverse decision bias as a function of pairs memory. Decision bias was operationalized as the difference in the tendency to choose rewarded items in chosen pairs and unchosen pairs in the Final Decisions phase, and memory was operationalized as accuracy in the memory test for the deliberation pairs. In Experiments 2 to 4 we also included a condition predictor and its interaction with memory (conditions 1 and 2 in Experiment 2: more and less repetitions, in Experiment 3: high and low reward, Experiment 4: same and different painter). For these experiments, we also computed the memory slope for each condition type separately. Columns represent the different coefficients, and cells depict median and 95% highest density intervals (HDIs) of the posterior distribution of each coefficient. HDIs that exclude zero are marked with an asterisk. Exp. = Experiment.

Supplementary Table 6. Choice memory and decision bias model regression coefficients.

Exp	Intercept	Memory	Condition	Memory x Condition
Pilot	0.23 [-0.01, 0.47]	0.33 [0.05, 0.61]*	-	-
Exp. 1	0.31 [0.16, 0.47]*	0.22 [0.04, 0.40]*	-	-
Exp. 2	0.23 [0.00, 0.47]*	0.26 [0.01, 0.52]*	-0.14 [-0.36, 0.08]	0.15 [-0.08, 0.40]
Exp. 3	0.29 [0.08, 0.48]*	0.20 [-0.03, 0.44]	0.04 [-0.15, 0.23]	-0.01 [-0.23, 0.21]
Exp. 4	0.11 [-0.07, 0.30]	0.40 [0.18, 0.63]*	-0.06 [-0.24, 0.12]	0.11 [-0.10, 0.33]

Exp.	Memory (Condition1)	Memory (Condition2)
Exp. 2	0.41 [0.08, 0.76]*	0.11 [-0.25, 0.47]
Exp. 3	0.19 [-0.13, 0.53]	0.21 [-0.10, 0.52]
Exp. 4	0.52 [0.23, 0.80]*	0.29 [-0.04, 0.63]

Note: In all experiments we ran a Bayesian linear regression predicting inverse decision bias as a function of choice memory. Decision bias was operationalized as the difference in the tendency to choose rewarded items in chosen pairs and unchosen pairs in the Final Decisions phase. Choice memory was operationalized as accuracy in the memory test asking participants which painting they chose during Deliberation phase (see more details in Supplementary Text 2). In Experiments 2 to 4 we also included a condition predictor and its interaction with memory (conditions 1 and 2 in Experiment 2: more and less repetitions, in Experiment 3: high and low reward, Experiment 4: same and different painter). For these experiments, we also computed the memory slope for each condition type separately. Columns represent the different coefficients, and cells depict median and 95% highest density intervals (HDIs) of the posterior distribution of each coefficient. HDIs that exclude zero are marked with an asterisk. Exp. = Experiment.

Supplementary Text

Supplementary Text 1 – Reaction times analysis in Experiment 1

We looked at reaction times as a proxy for value update. It is well known that choices for reward are faster than choices to avoid loss. Accordingly, we expect participants to be faster when they choose the more valuable painting. If participants did not update the value of unchosen pairs, reaction times for unchosen pairs should be similar, regardless of participants' choices. If however unchosen options were updated, we would expect reaction times for unchosen options to differ based on the experienced outcome of the their chosen counterpart. We first observed that reaction times in chosen pairs ($M = 0.80 \pm 0.01$ seconds) were faster than unchosen pairs ($M = 0.96 \pm 0.01$ seconds, see Supplementary Figure 1a), as expected. To assess how reaction times modulated choices within each pair type we ran a multilevel Bayesian logistic regression predicting the tendency to select gain items as a function of reaction times and pair type (chosen or unchosen pairs). We z-scored reaction times to account for individual variability in response times. We then rearranged the model coefficients to get a slope estimate predicting the effect of reaction times on choices for chosen and unchosen pairs separately. A positive slope would suggest that selecting no-gain items is accompanied with faster responses, and a negative slope would suggest that selecting no-gain items is accompanied with slower responses. We found that the slope for unchosen pairs was substantially positive (ß = 0.10 [0.05, 0.15], see Supplementary Figure 1b), suggesting that participants viewed unchosen items previously paired with unrewarded items as more valuable than those paired with rewarded items. Reaction times did not significantly modulate choices in chosen pairs (ß = -0.07 [-0.21, 0.08], see Supplementary Figure 1b), possibly because the tendency to select gain items was very high across participants so there was not enough variability in the dependent measure. We observed the same effects when we used raw reaction times rather than normalized ones (slope unchosen: ß = 0.33 [0.17, 0.49], slope chosen: ß = 0.02 [-0.43, 0.53]). Note that the reaction times analysis was exploratory and was not pre-defined in our preregistration on Open Science Framework (https://osf.io/chsvw).

Supplementary Text 2 – Analysis of the Surprise Memory phase

In the Surprise Memory test we presented pairs of paintings and asked participants whether each pair was an intact pair from the Deliberation phase or a recombined pair (pairs that included a chosen painting and an unchosen painting which did not appear together during Deliberation). If participants responded "intact", we also asked them which of the two paintings they chose during Deliberation. Overall, participants remembered the pairs above chance in all experiments (pairs memory accuracy across experiments: 0.63 ± 0.01, see Supplementary Table 1). To determine whether participants remembered their chosen items regardless of whether they remembered the pairings from Deliberation, we computed an overall choice accuracy score across hit and false alarm trials. Participants were above chance in remembering their choices (choice memory accuracy across experiments: 0.84 ± 0.01, see Supplementary Table 1). We then asked whether choice accuracy predicted decision bias in the Final Decisions phase. To this end, we ran a Bayesian linear regression predicting inverse decision bias (mean tendency to select gain in chosen pairs minus unchosen pairs) as a function of choice memory accuracy. In Experiment 2 to 4 an additional predictor was included to account for the different conditions. We found that choice memory predicted inverse bias in Experiment 1 and the Pilot study (see Supplementary Table 6). But unlike pairs memory, choice memory did not predict inverse bias in all conditions in Experiment 2 to 4 (see Supplementary Table 6 and 5).

Supplementary Text 3 – Pilot study and Experiments 2 to 4

Pilot study

This exploratory experiment tested our initial hypotheses about value inference of unchosen options.

Method. 93 Mechanical Turk (MTurk) participants took part in the study (mean age: 28.65 ± 4.03, 36 females, 57 males) and additional 7 participants were excluded from analyses because they met one of our pre-defined exclusion criteria (see Method section in the main text). The base payment was $3 with an average (and standard deviation) bonus earnings of $2.62 ± 0.53.

The experiment was essentially identical to Experiment 1 with the following exceptions: (1) The experiment did not include the Post-task Ratings and the Outcome Estimation phases, (2) the Deliberation phase included 10 pairs (instead of 12), and (3) the Final Decisions phase included four blocks with 50 trials each (25 unique chosen pairs and 25 unique unchosen pairs, randomly intermixed; instead of 3 blocks with 72 trials each in Experiment 1).

Results. In the Final Decisions phase participants tended to choose rewarded items in chosen pairs, but unrewarded ones in unchosen pairs (see Supplementary Tables 1-3). This inverse decision bias was predicted by recognition memory of the deliberation pairs (see Supplementary Tables 1 and 5).

Experiment 2

The goal of Experiment 2 was to manipulate memory encoding of the deliberation pairs by changing the number of times the pairs were repeated in the Deliberation phase.

Method. The experiment included 96 MTurk participants (mean age: 28.70 ± 4.54, 46 females, 49 males, 1 other), with additional 6 participants who were excluded from analyses. The base payment was $3.5 with an average bonus of $3.19 ± 0.66.

The experiment was similar to Experiment 1 with the following exceptions: (1) we introduced two types of deliberation pairs, (2) we used 60 representational paintings depicting only people (not objects or landscapes), and (3) there was no Outcome Estimation phase.

The Deliberation phase included 16 pairs, half of which repeated six times and the other half repeated twice (Supplementary Figure 2, left), for a total of 64 trials. As in Experiment 1, participants were told they will practice the decisions several times before committing to their final choice (in the last deliberation block). Yet instead of three blocks, the Deliberation phase included six blocks. The eight high-repeated pairs were presented in each of the first five blocks (in a random order), and the remaining low-repeated pairs were scattered across these five blocks, with no repetition. The last block included all pairs, randomly ordered. For every pair, participants were asked to explain their choice once throughout the first five blocks. Participants were not informed about the two pair types, and the pairs were not marked on the screen in any particular manner.

To compare performance between the two deliberation conditions we adapted the Outcome Learning, Final Decisions and Surprise Memory phases in the following manner. In the Outcome Learning phase, half of the chosen paintings in each deliberation condition were assigned to be

rewarded (as in Experiment 1, they were centered at $150 with standard deviation of $10) and the other half to be unrewarded ($0 earnings). Each chosen painting was presented six times, for a total of 96 trials. In the Final Decisions phase, the decision pairs were constructed within each deliberation condition, by creating all possible combinations of rewarded and unrewarded paintings within a condition. This resulted in 16 unique chosen pairs and 16 unique unchosen pairs for each condition, and a total of 72 pairs for both conditions. The Final Decisions phase included four blocks, each with the 72 unique pairs, randomly ordered, for a total of 288 trials. Finally, in the Surprise Memory phase, the recombined pairs were constructed within each deliberation condition and they included a chosen and an unchosen paintings that were not paired together during the deliberation phase but were from the same deliberation condition. The Surprise Memory phase included 16 recombined and 16 intact pairs, randomly ordered.
Results. To assess whether our memory manipulation was successful we looked at the Surprise Memory phase. Indeed, participants were more accurate in the recognition memory test for high-repeated pairs compared to the low-repeated pairs (see behavioral performance in Supplementary Table 1; multilevel logistic regression predicting accuracy by condition type: $ß_{intercept} = 0.55$ [0.45, 0.66]; $ß_{condition} = 0.14$ [0.06, 0.22]). We then tested whether this memory boost modulated decisions in the Final Decisions phase. Participants tended to choose unrewarded items for unchosen pairs in the high-repeated condition but not in low-repeated condition (see behavioral performance in Supplementary Table 1 and unchosen intercepts coefficients in Supplementary Table 3). However, the difference between the two conditions was not substantial (probability to select $S_{unchosen}+$ in low minus high repetition: 0.02 ± 0.03; unchosen intercept difference: $ß = 0.07$ [-0.26, 0.41]). Importantly, in both conditions memory of the pairs predicted inverse decision bias, with no difference between the conditions (see Memory x Condition coefficient in Supplementary Table 5).

Experiment 3

The goal of Experiment 3 was to manipulate participants' motivation by changing the magnitude of potential earnings of the chosen paintings.
Method. The experiment included 95 MTurk participants (mean age: 28.39 ± 4.29, 49 females, 45 males, 1 other), with additional 8 participants excluded from analyses. The base payment was $3 with an average bonus of $4.32 ± 0.25. The design of the experiment was identical to Experiment 2, but it differed in the deliberation manipulation and the magnitude of reward.
The deliberation phase included 16 pairs, half of which were marked as high-stakes pairs, and the other half as low-stakes pairs (Supplementary Figure 2, center). Participants were explicitly told that the paintings came from two types of galleries: a *high-stakes* gallery that can produce high profits (centered around $1500 and standard deviation of $100) and a *low-stakes* gallery that can produce low profits (earnings centered around $150 and standard deviation of $10). The gallery stakes were meant to incentivize participants, as they were told that one of their decisions will be played out for real and they will receive 0.2% of the auction profits for their chosen painting. If this was a high-stake painting that resulted in a gain, they could earn around $3 of bonus money,

as opposed to $0.3 for a low-stake painting. The two gallery types were marked on the screen using an orange (high-stakes) and a blue (low-stakes) frame around the deliberation pair (Supplementary Figure 2, center). As in Experiment 1, the Deliberation phase had three blocks, each included all deliberation pairs, randomly ordered. Half of the chosen paintings in each deliberation condition were assigned as rewarded paintings (high-stakes: centered at $1500 with standard deviation of 100; low-stakes: centered at $150 with standard deviation of 10) and the other half as unrewarded paintings ($0 earnings). At the end of the Outcome Learning phase, all participants were informed that the trial that was played for real included a high-stakes painting that resulted in a gain of $1500, affording them extra $3. As in Experiment 2, the pairs in the Final Decisions phase and the recombined pairs in the Surprise Memory phase were constructed within each deliberation condition. In the Final Decisions and Surprise Memory phases, the within-condition pairings were not marked in any way on the screen and participants were not informed about these pairings.

Results. To assess whether participants were more motivated in the high-stakes gallery condition we looked at their performance for chosen pairs in the Final Decisions phase. Indeed, participants tended to choose rewarded items more frequently in the high-stakes compared to the low-stakes condition (see behavioral performance in Supplementary Table 1; chosen intercept difference [high- minus low-stakes]: $ß = 0.64$ [0.23, 1.09]), suggesting they learned the new values of high-stakes paintings better than the low-stakes paintings. This motivation boost did not modulate memory of the deliberation pairs (see Supplementary Table 1 for means; multilevel logistic regression predicting memory accuracy by condition type: $ß_{intercept} = 0.59$ [0.47, 0.70]; $ß_{condition} = -0.01$ [-0.09, 0.07]). Interestingly, as in Experiment 2, participants tended to choose unrewarded items in the high-stakes gallery condition, but not in the low-stakes gallery (see means in Supplementary Table 1 and unchosen intercepts coefficients in Supplementary Table 3), yet the difference between the conditions was not substantial (probability to select $S_{unchosen}+$ in low- minus high- stakes: 0.02 ± 0.03; unchosen intercept difference: $ß = 0.21$ [-0.14, 0.56]). Furthermore, in both conditions memory of the pairs predicted decision bias, with no difference between the conditions (see Memory x Condition coefficient in Supplementary Table 5).

Experiment 4

The goal of Experiment 4 was to manipulate the strength of the high-level association of pairs of paintings. To this end, we told participants that some pairs involved art work painted by the same painter, while others were painted by two different painters. To increase the visual binding of the same-painter paintings, we presented them on top of a mutual white frame, whereas the different-painter paintings were presented on top of two separate white frames. We assumed that both the semantic cue (painter origin) and the visual cue (white frames) would modulate the relational encoding of the pairs.

Method. The experiment included 93 MTurk participants (mean age: 27.95 ± 4.05, 48 females, 45 males), with additional 6 participants excluded from analyses. The base payment was $3.5 with an average bonus of $3.06 ± 0.78.

The design of the experiment was identical to Experiment 3 with the exception that it did not include the Post-task Rating phase and the Deliberation phase included a different manipulation. The deliberation phase included 16 pairs, half of which were marked as same-painter pairs and the other half as different-painter pairs (Supplementary Figure 2, right). We ran two versions of the experiment. In Experiment 4a (n = 45, additional 4 were excluded), paintings reflected their condition type, such that for same-painter condition we used paintings from the actual same painter and vice versa for the different-painter condition. To make sure our results do not stem from potential visual similarities between paintings of the same painter, in Experiment 4b (n = 48, additional 2 were excluded) we shuffled the paintings but used the same instructions. The two experiments yielded similar results so we collapsed them into a single experiment. The Deliberation phase included three repetitions and chosen paintings could either be rewarded (centered at $150 with standard deviation of 10) or unrewarded ($0 earnings) in the Outcome Learning phase. The Final Decisions and Surprise Memory phases were identical to Experiments 2 and 3.

Results. The binding manipulation modulated the pairs memory. Pairs marked as painted by the same painter were remembered better than those by a different painter (see behavioral performance in Supplementary Table 1; multilevel logistic regression predicting memory accuracy by condition type: $ß_{intercept} = 0.54$ [0.39, 0.69]; $ß_{condition} = 0.20$ [0.10, 0.30]). Interestingly, the memory advantage of the same-painter condition also modulated choices in the Final Decisions phase condition (see Memory X Condition coefficient in Supplementary Table 5). The effect of memory on inverse decision bias was stronger for same versus different painter conditions (difference in memory slopes: ß = 0.60 [0.19, 1.02]). Notably, as in Experiments 2 and 3, participants tended to choose unrewarded items in the same-painter condition, but not in the different-painter condition (see behavioral performance in Supplementary Table 1 and unchosen intercepts coefficients in Supplementary Table 3), yet the difference between the conditions was not substantial (probability to select $S_{unchosen}$+ in different minus same painter: 0.06 ± 0.04; unchosen intercept difference: ß = 0.24 [-0.15, 0.62]).
