# Supplementary material for: Memory and decision making interact to shape the value of unchosen options
Source: Nat Commun. 2021 Jul 30;12:4648. doi: 10.1038/s41467-021-24907-x (PMC8324852; doi:10.1038/s41467-021-24907-x)
Supplement: Supplementary file 3 — Reporting Summary [file 41467_2021_24907_MOESM3_ESM.pdf]

nature research

Corresponding author(s): Natalie Biderman and Daphna Shohamy

Last updated by author(s): Jun 11, 2021

Reporting Summary

Nature Research wishes to improve the reproducibility of the work that we publish. This form provides structure for consistency and transparency in reporting. For further information on Nature Research policies, see Authors & Referees and the Editorial Policy Checklist.

Statistics

For all statistical analyses, confirm that the following items are present in the figure legend, table legend, main text, or Methods section.

n/a	Confirmed	
☐	☒	The exact sample size (n) for each experimental group/condition, given as a discrete number and unit of measurement
☐	☒	A statement on whether measurements were taken from distinct samples or whether the same sample was measured repeatedly
☐	☒	The statistical test(s) used AND whether they are one- or two-sided<br>*Only common tests should be described solely by name; describe more complex techniques in the Methods section.*
☐	☒	A description of all covariates tested
☐	☒	A description of any assumptions or corrections, such as tests of normality and adjustment for multiple comparisons
☐	☒	A full description of the statistical parameters including central tendency (e.g. means) or other basic estimates (e.g. regression coefficient) AND variation (e.g. standard deviation) or associated estimates of uncertainty (e.g. confidence intervals)
☐	☒	For null hypothesis testing, the test statistic (e.g. F, t, r) with confidence intervals, effect sizes, degrees of freedom and P value noted<br>*Give P values as exact values whenever suitable.*
☐	☒	For Bayesian analysis, information on the choice of priors and Markov chain Monte Carlo settings
☐	☒	For hierarchical and complex designs, identification of the appropriate level for tests and full reporting of outcomes
☐	☒	Estimates of effect sizes (e.g. Cohen's d, Pearson's r), indicating how they were calculated

Our web collection on statistics for biologists contains articles on many of the points above.

Software and code

Policy information about availability of computer code

Data collection	Data collection was conducted online in Mechanical Turk. Codes were programmed in javascript using the JSPsych library version 6.0.5.
Data analysis	All data analysis was completed in R (version 3.5.2) ,using RStudio (version 1.1.463). Bayesian models were performed using “rstanarm” package (version 2.18.2). To determine the sample size of Experiment 1, we computed the effect size of the Pilot study. To this end, we used "lme4" (version 1.1-19), "pwr" (version 1.2-2) and "lsr" (version 0.5) packages.

For manuscripts utilizing custom algorithms or software that are central to the research but not yet described in published literature, software must be made available to editors/reviewers. We strongly encourage code deposition in a community repository (e.g. GitHub). See the Nature Research guidelines for submitting code & software for further information.

Data

Policy information about availability of data

All manuscripts must include a data availability statement. This statement should provide the following information, where applicable:

- Accession codes, unique identifiers, or web links for publicly available datasets
- A list of figures that have associated raw data
- A description of any restrictions on data availability

Behavioral data generated and analysed during the current study are available in GitHub with the identifier (data DOI: http://doi.org/10.5281/zenodo.4926569). Source data are provided with this paper.

Field-specific reporting

Please select the one below that is the best fit for your research. If you are not sure, read the appropriate sections before making your selection.

☐ Life sciences ☒ Behavioural & social sciences ☐ Ecological, evolutionary & environmental sciences

For a reference copy of the document with all sections, see nature.com/documents/nr-reporting-summary-flat.pdf

Behavioural & social sciences study design

All studies must disclose on these points even when the disclosure is negative.

Study description	The study includes 5 quantitative experiments, all of which are within-subject design.
Research sample	All experiments included online participants from Mechanical Turk residing within the United States. We added a restriction on Mechanical Turk to include participants within the age range of 19 to 36, and approval rate of above 90%. Working with online participants allowed us to engage a large number of diverse respondents outside of a university subject pool, allowing for potentially a more representative sample. However, we did not collect demographic information other than age and sex so we cannot tell how representative was the sample. Participants were compensated for their participation. Demographic information: Pilot: n=93, mean age: 28.65 ± 4.03, 36 female, 57 male. Experiment 1: n=235, mean age: 28.54 ± 4.49, 132 female, 101 male, 2 other. Experiment 2: n=96, mean age: 28.70 ± 4.54, 46 female, 49 male, 1 other. Experiment 3: n=95, mean age: 28.39 ± 4.29, 49 female, 45 male, 1 other. Experiment 4: mean age: 27.95 ± 4.05, 48 female, 45 male.
Sampling strategy	Sample size of the exploratory Pilot study was determined to be n=100, including the excluded participants, following previous online experiments conducted in our lab. Experiments 2 to 4 followed the rationale of the Pilot study. Experiment 1 was preregistered on Open Science Framework and was meant to replicate the main findings of the Pilot study. Following Simonsohn, (2015), we defined the sample size of Experiment 1 to be 2.5 times the sample size of the Pilot study, which is 235 participants (we calculated 2.5 times the original sample size of 93 participants and rounded up by a few). A sample size of 235 participants gave us above 99% power to detect the effect size of the Pilot study, computed based on a simplified version of our main logistic regression model. Namely, we fitted a logistic function for each participant using "lme4" package and then tested the unchosen intercept coefficients against zero ($t(92) = -3.71$; $p < 0.001$; Cohen's d = 0.39; power analysis was performed using "pwr" package; and Cohen's d was computed using "lsr" package).
Data collection	Data collection in all experiments was conducted online, with participants recruited from Amazon's Mechanical Turk. The experiments required participants to respond using their keyboard and mouse. The researchers were not blind to the study's hypothesis and experimental conditions.
Timing	Pilot: conducted between June 2019 and July 2019.<br>Experiment 1: conducted on April 2020<br>Experiment 2: conducted on November 2019<br>Experiment 3: conducted on between November 2019 and January 2020<br>Experiment 4: conducted on July 2019
Data exclusions	Because the study included online participants, we applied the following exclusion criteria which were all aimed to ensure that participants were attending the task (see their preregistration on Open Science Framework; https://osf.io/chsvw): (1) Below chance performance (probability to choose gain items below 0.5) in the final decisions phase for chosen pairs, indicating participants who did not learn the new values of selected paintings (for Experiments 2 to 4, the exclusion performance is computed across conditions); (2) More than 25 missed responses in either the outcome learning phase (where participants had to register the outcomes they observed) or the final decisions phase; (3) More than 25 events where participants were browsing a different window in any experimental phase (blur-focus events detected using jsPsych library; de Leeuw, 2015); (4) More than 10 trials in the deliberation phase where responses were too fast (below 300 msec; these trials were accompanied with a warning), signifying no actual deliberation; and (5) More than 10 failed attempts to answer a comprehension quiz administered after instructions in any experimental phase. Participants who met at least one of these exclusion criteria was removed from all analyses.
Non-participation	No participants dropped out or declined.
Randomization	There was no group allocation because all experiments had a within-participant design. Relevant conditions within every experiment were randomly assigned when possible (see more details in the Method section of the experiments).

Reporting for specific materials, systems and methods

We require information from authors about some types of materials, experimental systems and methods used in many studies. Here, indicate whether each material, system or method listed is relevant to your study. If you are not sure if a list item applies to your research, read the appropriate section before selecting a response.

Materials & experimental systems

n/a	Involved in the study
☒	☐ Antibodies
☒	☐ Eukaryotic cell lines
☒	☐ Palaeontology
☒	☐ Animals and other organisms
☐	☒ Human research participants
☒	☐ Clinical data

Methods

n/a	Involved in the study
☒	☐ ChIP-seq
☒	☐ Flow cytometry
☒	☐ MRI-based neuroimaging

Human research participants

Policy information about studies involving human research participants

Population characteristics	See "Research sample" above
Recruitment	Participants were recruited from Amazon's Mechanical Turk platform. The recruitment was restricted to include participants residing within the US, within the age range of 19 to 36, and approval rate of above 90%. All participants were compensated for participation. Because online participants choose to opt in the study, our results might be affected by self-selection bias.
Ethics oversight	Institutional Review Board (IRB) at Columbia University through Columbia IRB Protocol #AAAI1488

Note that full information on the approval of the study protocol must also be provided in the manuscript.
