# Supplementary material for: Memory and decision making interact to shape the value of unchosen options
Source: Nat Commun. 2021 Jul 30;12:4648. doi: 10.1038/s41467-021-24907-x (PMC8324852; doi:10.1038/s41467-021-24907-x)
Supplement: Supplementary file 4 — Source data [file 41467_2021_24907_MOESM4_ESM.zip › Source_Data/README.html]

README.utf8.md


### Source Data – How to use


#### Natalie Biderman and Daphna Shohamy, May, 2021


The figures and tables presented in the manuscript can be reproduced using the R Markdown script “create\_figures\_and\_tables\_from\_source\_data.Rmd” which takes the excel data file “Source\_Data.xlsx” as input. In the latter file, we separate the data associated with every subplot and table into different sheets. Below we explain how to read and use the excel file.

#### Figures

Bar and scatter plots include trial-averaged data points of individual participants. We also included the data to reproduce the model fit plots presented in the figures. All of our models are Bayesian generalized linear models. As such, model coefficients are represented using samples from their approximate posterior distribution. The model fits illustrate the median and 95% highest density interval (HDI) of the approximate posterior distribution. We used the following procedure to plot the model fits. First, we generated a sequence of values on the x-axis to cover the entire range of observed data (e.g., for a model predicting inverse decision bias as a function of pairs memory presented in Figure 3b, we created 100 values of pairs memory ranging from 0.33 to 1). We then predicted the y value of each of these x points using all samples (12,000) from the posterior distribution of the coefficients of interest (e.g., for every sample we computed y = intercept + slope \* x). This resulted in a 12,000 by 100 (samples by x-values) dimensional array of predictions. Lastly, we computed the median and the 95% HDI of the 12,000 predicted values. Accordingly, excel sheets that present data for model fits include the following columns: (1) obs – the index of the x-axis observation, (2) median – median of the distribution of every observation, (3) upper - the upper bound of the 95% HDI, (4) lower – the lower bound of the %95 HDI, (5) the x values (the column name matches the figure), (6) the predicted y values for every observation (the column name matches the figure) – this value is identical to the median.

#### Tables

Table 1 includes averages of behavioral metrics reported in the manuscript. Tables 2 to 6 present median and 95% HDI of different models. To reproduce these coefficients we included the posterior samples drawn from our Bayesian models.
